# The conventional diagnosis challenge: Real-time PCR and nested PCR correlation with the scoring system for individuals at high-risk of *Pneumocystis jirovecii* pneumonia

**DOI:** 10.7705/biomedica.7020

**Published:** 2023-08-31

**Authors:** Fernando Almeida-Silva, Rodrigo Almeida-Paes, Lisandra Serra-Damasceno, Edwiges Motta-Santos, Luiz Claudio Ferreira, Leonardo Pereira-Quintella, Marcela de Faria Ferreira, Mauro de Medeiros-Muniz, Rosely M. Zancopé-Oliveira

**Affiliations:** 1 Laboratório de Micologia, Instituto Nacional de Infectologia Evandro Chagas, Fundação Oswaldo Cruz, Rio de Janeiro, Brasil Instituto Nacional de Infectologia Evandro Chagas Fundação Oswaldo Cruz Rio de Janeiro Brasil; 2 Hospital São José de Doenças Infecciosas, Fortaleza, Ceará, Brasil Hospital São José de Doenças Infecciosas Ceará Brasil; 3 Departamento de Saúde Comunitária, Faculdade de Medicina, Universidade Federal do Ceará, Fortaleza, Ceará, Brasil Universidade Federal do Ceará Universidade Federal do Ceará Fortaleza Ceará Brazil; 4 Laboratório de Medicina Intensiva, Instituto Nacional de Infectologia Evandro Chagas, Fundação Oswaldo Cruz, Rio de Janeiro, Brasil Instituto Nacional de Infectologia Evandro Chagas Fundação Oswaldo Cruz Rio de Janeiro Brasil; 5 Serviço de Anatomia Patológica, Instituto Nacional de Infectologia Evandro Chagas, Fundação Oswaldo Cruz, Rio de Janeiro, Brasil Instituto Nacional de Infectologia Evandro Chagas Fundação Oswaldo Cruz Rio de Janeiro Brasil; 6 Serviço Ambulatorial do Instituto Nacional de Infectologia Evandro Chagas, Fundação Oswaldo Cruz, Rio de Janeiro, Brasil Instituto Nacional de Infectologia Evandro Chagas Rio de Janeiro Brasil

**Keywords:** Pneumonia, pneumocystis, fluorescent antibody technique, direct, real-time polymerase chain reaction, neumonía por *Pneumocystis*, técnica de inmunofluorescencia directa, reacción en cadena en tiempo real de la polimerasa

## Abstract

**Introduction.:**

*Pneumocystis jirovecii* is an opportunistic fungus that affects mainly people living with HIV (CD4 cell count lower than 200 cells/ml) and other immunosuppressed patients. Since P. *jirovecii* does not grow on routine mycological media, diagnosis of P. *jirovecii* pneumonia relies on indirect evidence of its presence in respiratory samples. Objectives. To associate the results of direct immunofluorescence and two molecular methods with a score to predict P. *jirovecii* pneumonia in patients with AIDS.

**Materials and methods.:**

A prospective study was conducted with 40 patients. A respiratory sample collected before treatment was subjected to direct immunofluorescence using the Merifluor kit, to nested PCR targeting the mitochondrial large subunit ribosomal RNA, and to the VIASURE real-time PCR kit.

**Results.:**

These three techniques revealed *P*. *jirovecii* in 6, 12, and 15 samples, respectively. All positive samples by direct immunofluorescence were positive by nested PCR, and all positive samples by nested PCR amplified by real-time PCR. There was a statistically significant association between the *P. jirovecii* pneumonia score and the molecular methods. Two patients were early diagnosed and responded well to treatment.

**Conclusion.:**

Molecular methods, especially real-time PCR, are recommended for early diagnosis of *P. jirovecii* pneumonia in AIDS patients.

## Resumen

**Introducción.:**

*Pneumocystis jirovecii* es un hongo oportunista que afecta principalmente a personas con HIV (recuento de CD4 menor de 200 células/ml) y a otros pacientes inmunosuprimidos. Como *P. jirovecii* no crece en los medios micológicos de rutina, el diagnóstico de neumonía por *P. jirovecii* se basa en la evidencia presente en muestras respiratorias.

**Objetivos.:**

Asociar los resultados de la inmunofluorescencia directa y los de dos métodos moleculares con un puntaje para predecir la neumonía causada por *P. jirovecii* en pacientes con sida.

**Materiales y métodos.:**

Se realizó un estudio prospectivo de 40 pacientes. Se recolectó una muestra respiratoria antes del inicio de tratamiento y se sometió a una prueba de inmunofluorescencia directa con el kit Merifluor, una PCR anidada para la amplificación de la subunidad larga del ribosoma mitocondrial y una PCR en tiempo real usando el kit VIASURE.

**Resultados.:**

Estas tres técnicas evidenciaron la presencia de *P. jirovecii* en 6, 12 y 15 muestras, respectivamente. Todas las muestras positivas por inmunofluorescencia directa fueron positivas en la PCR anidada y todas las muestras positivas en la PCR anidada amplificaron por PCR en tiempo real. Se encontró una asociación estadística entre los valores de la neumonía causada por *P. jirovecii* y los métodos moleculares. Dos pacientes con diagnóstico temprano respondieron satisfactoriamente al tratamiento.

**Conclusión.:**

Se recomiendan los métodos moleculares, especialmente la PCR en tiempo real, para el diagnóstico temprano de neumonía causada por *P. jirovecii* en pacientes con sida.

Fungal pulmonary infections remain one of the most important health problems encountered in immunocompromised individuals ^1^. In general, mycoses became a common finding after the AIDS epidemic onset, with several fungi causing disease in this population ^1^. Among these fungi, *Pneumocystis jirovecii* (formerly known as *Pneumocystis carinii*), which has a worldwide distribution, causes fungal pneumonia considered an AIDS- defining disease ^2^. *Pneumocystis jirovecii* pneumonia also occurs in non- HIV-infected patients such as transplant recipients, individuals with prolonged immunosuppressive therapies, with autoimmune complications, and neoplasic disorders under chemotherapy ^3^.

Historically, it was believed that *P. jirovecii* pneumonia was a direct consequence from a latent prior infection ^4^, but a study of 15 lung autopsies of immunocompetent individuals aged 15-75 years showed the absence of *P. jirovecii* and disproved this hypothesis ^5^. Nowadays, the most accepted hypothesis is *P. jirovecii* transmission from person to person or acquisition from the environment, both by airborne route. Furthermore, disease development can also occur due to reactivation or reinfection ^6^. This fungus can cause an asymptomatic infection to fulminant pneumonia depending on the host’s immune status. The most severe case of this disease presents when CD4+ lymphocyte count is lower than 200 cells/ml, frequently occurring in male patients ^7^.

The *P. jirovecii* pneumonia diagnosis is difficult, mainly because *P. jirovecii* cannot grow in traditional mycological culture media such as Sabouraud or Mycosel ^8^. Cultures of *P. jirovecii* are obtained only in pneumocyte cell cultures, which are not available in most laboratories ^9^. Histopathologic diagnosis by staining methods or fungal visualization (with the fluorescent dye calcofluor white) depends on the fungal burden, specific staining and the observer’s expertise ^10^. In some cases, the traditional histopathologic diagnosis can be difficult, due to similar structures present in the material that can lead to a misdiagnosis with other microorganisms, making differential diagnosis strongly necessary ^11^. In addition, serological methods based on antibody detection have limitations in *P. jirovecii* pneumonia diagnosis. Humans produce antibodies against the gpA protein, used in serological studies ^12^. However, due to high genetic variation, this protein is not widely studied. Another serological tests limitation is that patients from different geographic regions may have different levels of immune response to this surface protein ^13^.

Laboratory findings are generally not helpful in the *P. jirovecii* pneumonia diagnosis because many findings are compatible with other infections. For example, increased serum lactate dehydrogenase is present in HIV-infected patients ^14^^-^^16^. Symptoms such as fever, cough, dyspnea, and, in severe cases, respiratory failure, are also manifestations of other infections, such as tuberculosis ^17^, histoplasmosis ^18^, cryptococcosis ^19^, community- acquired pneumonias (CAP) ^20^, and more recently, coronavirus disease 2019 (COVID-19) ^21^.

Several research groups reported the use of molecular methods to detect and diagnose *P. jirovecii*^22^^-^^25^. Molecular methods have a high sensitivity and specificity, almost without cross-reactivity, and present better results when compared with traditional diagnostic methods, including direct immunofluorescence ^23^ and serology ^24^. Up to now, nested PCR and realtime PCR are the most widely used molecular diagnostic methods, and have different levels of sensitivity and specificity, according to the protocol and the selected target ^26^. These methods are not able to differentiate between colonization and infection, but provide fast and accurate results, important factors because faster results imply quicker diagnosis and treatment, improving the health quality of immunocompromised populations.

Even with a multitude of available molecular tests for *P. jirovecii* pneumonia diagnosis, the immunofluorescence assay remains as the gold standard for the diagnosis of pneumocystosis, as it detects the presence of spore or ascus (formerly known as cysts and trophozoites) ^27^. However, as exposed, it is a test with several limitations and requires careful result analysis, especially in cases with low fungal load, which can easily present false negative results with this technique ^23^. In these cases, it is hard to interpret if a positive molecular method result (performed with the same respiratory sample) is a true false-positive or if it indicates a fungal burden below the immunofluorescence detection limit.

This study aimed to compare the results of traditional, direct immunofluorescence assay versus molecular methodologies (nested PCR and a commercial real-time PCR kit) to detect *P. jirovecii* in respiratory samples from immunocompromised patients with fungal pneumonia suspicion and correlate with the scoring system to predict *P. jirovecii* pneumonia ^28^.

This study was approved by the Research Ethics Committee of the Evandro Chagas National Institute of Infectious Diseases - Oswaldo Cruz Foundation (INI-Fiocruz), CAAE 00580.0.009.000-09.

## Materials and methods

### Patients and samples

A study was conducted (2013-2014) in patients with suspected fungal pneumonia at Instituto Nacional de Infectologia Evandro Chagas, Fundação Oswaldo Cruz, Rio de Janeiro, Brazil. Inclusion criteria were patients over 18 years old and were hospitalized. The included patients were people living with HIV, a CD4+ cell count lower than 200 cells/ml, and clinical symptoms compatible with fungal pneumonia. Patients without sufficient respiratory samples to carry out all proposed methodologies were excluded. Respiratory samples for diagnosis were collected depending on the patient’s condition and consent. Samples included spontaneous or induced sputum, tracheal wash, and bronchoalveolar lavage in a convenience sampling.

### Culture

The culture was performed to check other possible fungal agents causing coinfections. After removing the airway mucus layer with citrate buffer and the N-acetylcysteine (a mucolytic agent) the samples were centrifuged at 11.500g for five minutes and cultivated in 2% Sabouraud Dextrose and Mycosel culture media (Becton Dickinson). Cultures were observed on a weekly basis during four weeks of incubation at room temperature. Possible fungi were identified by conventional mycologic techniques.

### Hematoxylin and eosin, Grocott’s and calcofluor white stains

Previous treatment with cellblock fixative was done for hematoxylin and eosin, and Grocott’s stain, to yield a cellular pellet from the respiratory materials in 1.5 ml. All samples were paraffin-embedded for posterior sections measuring 3-4 µm. The sections were stained with the traditional protocol for hematoxylin and eosin to evidence samples’ cellular characteristics. Moreover, for the Grocott's method, the samples were stained with metenamine silver nitrate and counterstained with light green. The calcofluor staining was performed using a commercial reagent (Calcofluor White Stain - Fluka Inc.). The reaction was performed using one drop of the sample, one drop of 10% potassium hydroxide, and one calcofluor drop, as the manufacturer instructed.

### Direct immunofluorescence

One ml of 0.1% dithiothreitol was added, as a mucolytic agent, to 1 ml of the respiratory specimens. The mixture was incubated at 37 °C for 15 minutes and the supernatant was discarded. After this step, the direct immunofluorescence assay was conducted using the Merifluor *Pneumocystis* kit (Meridian Biosciences, Inc, OH, USA) according to the manufacturer’s instructions. The results were observed with the fluorescence microscope Olympus-BX40® with a 400-fold magnification.

### DNA extraction

The DNA was extracted from the respiratory samples using the QIAamp® DNA mini kit (Qiagen, Hilden, Germany), following all the manufacturer's recommendations. DNA concentrations were estimated using the Nanovue™ Plus Spectrophotometer (GE Healthcare, Buckinghamshire, UK).

### Human housekeeping gene β-globin PCR and nested PCR for P. jirovecii

The single step polymerase chain reaction (PCR) for human housekeeping gene β-globin was performed using the primer pair βglobinF (5’-GCAAGA AAG TGC TCG GTG C-3’) and βglobinR (5’-TCA CTC AGT GTG GCA AAG GTG- 3’). The total reaction volume was 50 µl, using 10 µl of DNA, 10 mM Tris-HCI, 50 mM KCI, 2.5 mM MgCI_2_, 1.5 units of Taq DNA polymerase (ThermoFisher, MA, EUA), 30 pMol of each primer and 200 µM of dNTPs (ThermoFisher, MA, EUA). The PCR was conducted with the following conditions: 95 °C for 5 minutes; 45 cycles at 95 °C for 30 s, 57 °C for 30 s, 72 °C for 30 s and one final extension at 72 °C for 10 minutes ^29^.

The nested PCR targeting the *P. jirovecii* mitochondrial large subunit ribosomal RNA (mtLSUrRNA), was performed using in the first round the primers pAZ 102-H (5’- GTG TAC GTT GCA AAG TAC TC-3’) and pAZ 102-E (5’-GAT GGC TGT TTC CAA GCC CA-3’), and in the second reaction, the primers pAZ 102-X (5’-GTG AAA TAC AAA TCG GAC TAG G-3’) and pAZ 102-Y (5’-TCA CTT AAT ATT AAT TGG GGA GC-3’) ^18^. A total volume of 50 µl was set for each reaction, containing 10 µl DNA, 10 µM Tris-HCI, 50 µM KCI, 2.5 mM MgCI_2_,1.5 units of Taq DNA polymerase, 10 pM of each primer and 200 pM of dNTPs. The mix of the nested PCR was similar, except for the use of 2 µl of the first reaction, used as template for the second reaction. Both reactions were performed following the protocol previously described ^13^, with minor modifications: The first reaction was cycled 40 times at 94 °C for 1.5 minutes, 60 °C for 1.5 minutes and 72 °C for 2 minutes. The second round was cycled 40 times at 94 °C for 1.5 minutes, 61.7 °C for 1.5 minutes and 72 °C for 2 minutes ^30^.

Both reactions were performed in a Cl 000 thermalcycler (BioRad, Germany) and electrophoresed on a 1% agarose gel stained with 0.5% ethidium bromide.

### Viasure real-time PCR

The VIASURE *Pneumocystis jirovecii* real time PCR detection kit (CERTEST BIOTEC, Spain) was designed for P. jirovecii diagnosis in respiratory samples. After DNA isolation, the identification of P. jirovecii was performed by the amplification of a mtLSUrRNA conserved region, using specific primers and a fluorescent-labelled probe (FAM). The reaction was performed according to manufacturer’s instructions in a Agilent AriaMX (Agilent Technologies, Santa Clara, USA) qPCR thermal cycler. The reactions consisted in a polymerase activation at 95 °C for 2 minutes and 45 cycles of annealing/extension at 60 °C for 50 s. The results were analized in the Aria MX software (version 1.7.1).

### Pneumocystis jirovecii pneumonia diagnosis

The final diagnosis of *P. jirovecii* pneumonia was reached after the correlation of all test results and its classification based on the scoring system proposed by Smith, Forbes and Gazzard (1992) ^28^ (supplementary table 1). The response to sulphamethoxazole/trimethoprim treatment was also considered.

**Table 1:** Clinical-epidemiological characteristics of the 40 patients included in the study

Variable		n	%
Sex			
	Male	30	75
	Female	10	25
Age			
	18-19	1	2.5
	20-39	23	57.5
	40-59	16	40
Viral load^a^			
	Not available	19	47.5
	<1000	4	10
	1000-10.000	5	12.5
	10.001-100.000	2	5
	>100.001	10	25
CD4+ count^b^			
	<10	3	7.5
	11-50	7	17.5
	51-100	17	42.5
	101-200	13	32.5

^a^ copies/ml

^b^ cells/ml

### Statistical analysis

Concordance between the defined categories by the diagnostic methods (positive or negative) was evaluated using the kappa coefficient, interpreted as described ^31^. Statistical analyses were performed using GraphPad Prism (version 5.0) software. Non-parametric tests were used to compare groups of continuous variables. Categorical variables were analized using the Fisher exact test. A p-value less than 0.05 was considered significant.

### Institutional review board statement

The study was approved by the Institutional Ethics Committee of the Evandro Chagas National Institute of Infectious Diseases (protocol code CAAE 00580.0.009.000-09-date of approval: 26 February 2014).

## Results

### Patients and samples

During the study period, 40 patients fulfilled the inclusion criteria and were enrolled, resulting in 40 clinical respiratory samples (21 induced sputum, 11 tracheal wash, 5 bronchoalveolar lavage, and 3 spontaneous sputum) for analysis. Table 1 presents the clinical-epidemiological characteristics of the patients. After analyzing the clinical features of each patient, all individuals were assigned a final score to know the probability of having *P. jirovecii* pneumonia (table 2), ranging from 23 to 98%. All patients started sulphamethoxazole/ trimethoprim prophylaxis after the respiratory samples collection.

**Table 2.** Patients final score and *Pneumocystis jirovecii* pneumonia probability

Final scoring	Patients n (%)	***P. jirovecii* pneumonia probability (%)**
<-3	0 (0)	
-3 to +6	19(47.5)	23
> +6	10(25)	82-87
>+19	11 (27.5)	98

### Staining methods and culture

Most of the samples (72.5%) showed respiratory tract cells: squamous cells, cylindrical cells, and alveolar macrophages (data not shown) while 27,5% of the samples were paucicellular or presented cell absence. Among the staining techniques performed, calcofluor white, and silver staining did not reveal compatible P. jirovecii cells in any respiratory sample. All the cultures did not demonstrate any fungal growth. The results for all tested methodologies are presented in supplementary table 2.

### Direct immunofluorescence

**Figure 1.**
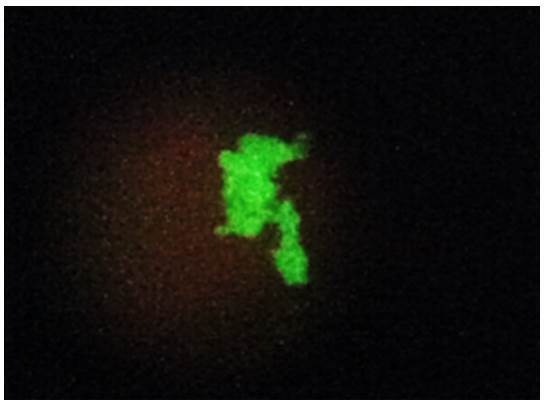
Representative positive direct immunofluorescence result at 400X. Fluorescent honeycomb structures seen in a sputum sample treated with the Merifluor® *Pneumocystis kit*

Among the 40 clinical specimens evaluated, six samples (15%) had structures morphologically compatible with P. *jirovecii* stained with a fluorescent green color and were considered positive by direct immunofluorescence using the Merifluor Pneumocystis kit (figure 1).

### Molecular methods

**Figure 2.**
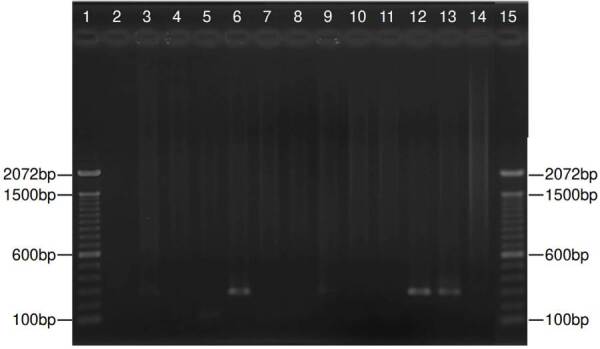
Representative nested polymerase chain reaction for jirovecii in 11 samples. Lanes 1 and 15: Molecular weight (1 kb plus DNA ladder - ThermoFisher Scientific, Inc.); lanes 3, 6, 9, and 12: Positive samples with a fragment of 260 bp; lanes 2, 4, 5, 7, 8,10 and 11: Negative samples; lane 13: Positive control, lane 14: Negative control. The size (base pairs - bp) of the bands is indicated in both sides.

**Figure 3.**
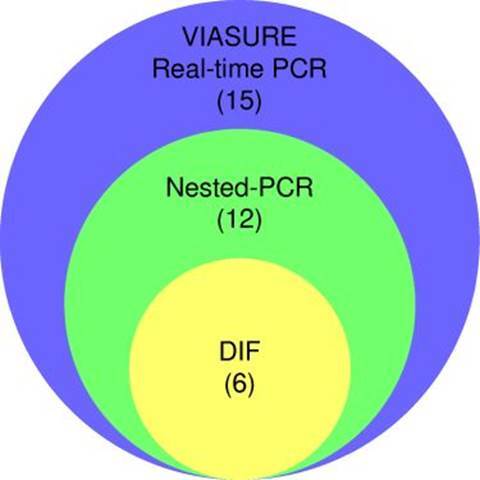
Venn diagram showing the number of positive results obtained in each method. An overlap of positive samples was observed with the different methodologies. DIF: Directimmunofluorescence; PCR: polymerase chain reaction

**Supplementary figure 1:**
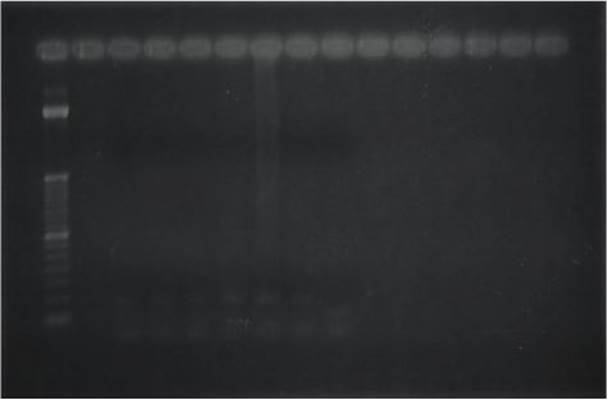
Representative image of the human (β -globin gene partial amplification in the studied samples

Partial amplification of the endogenous 0-globin control gene yielded a specific fragment of 79 bp, evidencing successful DNA extraction from all respiratory specimens (supplementary figure 1). Nested PCR targeting the mtLSUrRNA gene revealed 12 positive specimens (30%), with a 260 bp fragment amplification (figure 2). The VIASURE *Pneumocystis jirovecii* real-time PCR detection kit, designed for the same conserved region of mtLSUrRNA, amplified 15 samples (37.5%). All positive samples by direct immunofluorescence were also positive by nested PCR, and all positive samples by nested PCR were amplified by real-time PCR (figure 3).

### Clinical and laboratory findings correlation

The direct immunofluorescence, nested PCR, and VIASURE real-time PCR detection kit had discordant results for *P. jirovecii* detection. For this reason, a clinical-laboratory correlation with the results of all methodologies was performed, and generated a scoring system for individuals with high-risk of *P. jirovecii* pneumonia.

The direct immunofluorescence was negative for patients with a probability of 23% (19 patients among the 40 studied) for *P. jirovecii* pneumonia. This technique significantly demonstrated insatisfatory results in patients with low *P. jirovecii* pneumonia probability (p = 0.02, Fisher’s exact test).

Nested PCR results for patients with a 23% probability of having *P. jirovecii* pneumonia indicated only one positive (5%) and 18 negative samples. Among patients with 82% or higher probability for *P. jirovecii* pneumonia, 11 (52%) samples were positive, while 10 (48%) were negative.

A statistically significant difference between these two groups (p = 0.0015, Fisher’s exact test) was detected. The kappa index demonstrated a moderated correlation (0.583) between direct immunofluorescence and nested PCR (figure 3).

The results of VIASURE *Pneumocystis jirovecii* real-time PCR evidenced a statistically significant difference between these two groups (p = 0.0011, Fisher’s exact test); firstly, the group with 23% of PCP and in the second, the group higher than 82% of PCP. The kappa index demonstrated a moderated correlation (0.455) between direct immunofluorescence and real-time PCR results. In addition, an agreement was observed between nested PCR and real-time PCR (kappa = 0.833). All these results are summarized in table 3 and supplementary table 2.

**Table 3.** Positive results in the different *Pneumocystis jirovecii* diagnostic methods among four possible scores of high-risk individuals to *Pneumocystis jirovecii* pneumonia

***P. jirovecii* pneumonia probability (n)**	DIF		Nested PCR		VIASURE Real-time PCR	
	n	%	n	%	n	%
98% ^11^	2	18	6	54	7	63
87% ^2^	1	50	1	50	1	50
82% ^8^	3	37.5	4	50	5	62.5
23% ^19^	0	0	1	5	2	10

DIF: Direct immunofluorescence

## Discussion

This study evaluated the performance of methods for the diagnosis of pneumonia caused by *P. jirovecii*. The analyzed samples consisted of respiratory material collected from 40 patients with AIDS, with considerable immunosuppression and respiratory symptoms compatible with those presented by fungal diseases. The use of the scoring system proposed by Smith, Forbes and Gazzard (1992) in the included patients, allowed us to predict different probabilities of *P. jirovecci* pneumonia among them.

The gold standard method to detect P. jirovecii is direct immunofluorescence, since this technique detects *P. jirovecii* in its two lifecycle stages ^32^. Conventional techniques have shown intrinsical limitations, such as microscopic visualization of clinical samples which depends on cellular quality, microorganism amount, and observer expertise ^26^. In our sampling, the gold standard method showed the lowest positivity among the other diagnostic techniques tested. Direct immunofluorescence makes it possible to detect *P. jirovecii* only in patients with a higher probability of *P. jirovecii* pneumonia. It may indicate a failure of *P. jirovecii* pneumonia diagnosis in oligosymptomatic patients or at early stages of the disease. Our results corroborate the indication of other research groups about the use of molecular methods as alternative techniques to detect this fungal pathogen.

Several reports have shown the low sensitivity of conventional techniques when compared to molecular methods. A study with 275 clinical respiratory specimens, comparing Grocott's stain, direct immunofluorescence, and nested PCR (targeting the *mtLSUrRNA*) revealed nine positive samples by the Grocott's stain and 16 positive samples by direct immunofluorescence. However, when the samples were evaluated by nested PCR, the authors found *P. jirovecii* DNA in 44 samples ^26^.

Another report, using 50 clinical specimens of respiratory origin and comparing three methodologies: Giemsa, direct immunofluorescence, and nested PCR (targeting the mtLSUrRNA), showed that Giemsa stain detected *P. jirovecii* only in one sample, direct immunofluorescence yielded four positive results and nested PCR resulted in 36 positive samples ^33^.

A study comparing nine molecular methods showed that nested PCR targeting the *mtLSUrRNA* gene should be considered the most sensitive technique ^34^ for *P. jirovecii* pneumonia diagnosis. The explanation for the better performance of this technique is that the *mtLSUrRNA* gene is fully involved in basic metabolic processes, and it has a high degree of genetic conservation ^35^ in *P. jirovecii*. In addition, the high number of *P. jirovecii* mitochondrias makes the *mtLSUrRNA* gene an excellent target for *P. jirovecii* pneumonia diagnosis ^26^, increasing the sensitivity of the nested PCR. For this reason, this partial sequence has become the molecular target most suitable for *P. jirovecii* detection. The results herein corroborate the utility of the mtLSUrRNA gene for early detection of pneumonia caused by *P. jirovecii*^36^.

In contrast with most eukaryotic organisms, *P. jirovecii* has only one copy of the gene encoding the ribosomal RNA, and this explains why techniques that use internal transcribed spacer (ITS) region as diagnostic target has low efficiency ^37^. The VIASURE real-time PCR kit was also developed to target the *mtLSUrRNA* gene. The results obtained in this study were better than those of nested PCR, with three additional patients diagnosed with *P. jirovecii* pneumonia. These results suggest that the VIASURE real-time PCR should be used for *P. jirovecii* pneumonia diagnosis in laboratories with the structure to perform it.

The lack of studies with this commercial kit does not allow us to establish a difference between *P. jirovecii* colonization and infection. However, our results together with the proposed clinical score indicated a possible *P. jirovecii* infection in the evaluated patients. To the best of our knowledge, this is the first study associating the Smith *et al*. (1992) score with laboratory data. Positive samples were detected in patients with lower *P. jirovecii* pneumonia probabilities based on the scoring system, which leads us to believe that this detection occurred in the early stages of the disease. Among the 40 studied patients, we observed that nested PCR was positive in the 12 samples, and among these, six were also positive by direct immunofluorescence. The increase in the positivity rate associated with the scoring system made us conclude that molecular methods, although not new, remain useful tools for the *P. jirovecii* pneumonia diagnosis.

Nested-PCR is an accessible technique for many laboratories, and the results are not observer-dependent like those of direct immunofluorescence. However, cross-contamination may occur between the first and second reactions, so laboratories should be careful when handling these samples. Real-time PCR provides fast results, also observer-independent, and have a low chance of external DNA contamination. However, it requires expensive equipment that may not be available in all centers handling patients with AIDS.

With the results of this work, it was concluded that nested PCR provides accurate results in laboratory practice for pneumocystosis diagnosis, especially in laboratories with basic infrastructure. However, the VIASURE real-time PCR showed superior results compared to nested PCR. Therefore, we suggest that, in countries where the kit is approved for diagnostic use, it should be performed as a routine test when the necessary infrastructure is available. Molecular results should be interpreted along with each patient’s clinical signs and symptoms, and the score previously suggested ^28^ is adequate for this purpose.

## Archivos suplementarios

**Supplementary table 1.** *Pneumocystis jirovecii* pneumonia probability based on clinical features following a scoring system for high-risk individuals

Feature	Patients n	Score
Starting score	40	+3
Clinical symptoms		
Yes	9	+6
No	31	-9
No prophylaxis		
Yes	27	+7
No	13	-9
Infiltrates in tórax radiography		
Yes	26	+12
No	14	-6
Desaturation		
Yes	16	+9
No	24	-13
Range of possible scores		-34 to +37

**Supplementary table 2.** Individual results of the patients included in this study (specimen, T CD4+ count, viral load, direct immunofluorescence, nested-PCR, real-time PCR and *Pneumocystis jirovecii* pneumonia probability)

Sample number	Specimen	T-CD4+	VL	DIF	Nested PCR	VIASURE Real-time PCR	PCP Probability
1	TW	51	NA	0	0	0	<23%
2	TW	51	NA	0	0	0	98%
3	TW	51	NA	0	0	0	98%
4	TW	189	<40	0	0	0	>87%
5	TW	110	544.934	0	0	0	<23%
6	IS	305	2.427.730	0	1	1	>82%
7	TW	7	116.689	0	1	1	98%
8	IS	112	850.578	0	1	1	98%
9	IS	8	350.638	1	1	1	>82%
10	IS	200	10.745	0	0	0	<23%
11	IS	308	NA	0	0	0	<23%
12	IS	43	NA	0	0	0	<23%
13	IS	62	NA	0	0	0	<23%
14	IS	70	NA	0	0	1	<23%
15	IS	64	NA	0	0	0	<23%
16	SS	60	NA	1	1	1	>82%
17	BAL	402	333.849	0	1	1	<23%
18	IS	32	177.036	1	1	1	>82%
19	IS	39	NA	1	1	1	98%
20	TW	469	48	0	0	0	<23%
21	IS	42	NA	0	0	1	98%
22	IS	46	<40	0	0	0	<23%
23	IS	68	4.740	0	0	1	>82%
24	IS	70	NA	0	0	0	>82%
25	TW	67	NA	0	0	0	>82%
26	IS	98	45.904	0	0	0	>82%
27	IS	92	NA	0	0	0	<23%
28	BAL	39	3.193	0	0	0	<23%
29	TW	48	5.002	0	0	0	98%
30	SS	130	25.940	0	0	0	<23%
31	SS	86	580.521	0	0	0	<23%
32	IS	35	NA	0	0	0	98%
33	BAL	238	1.628	0	1	1	98%
34	TW	111	1.689	1	1	1	>87%
35	SS	0	718.648	1	1	1	98%
36	IS	64	NA	0	0	0	<23%
37	BAL	128	NA	0	0	0	<23%
38	BAL	238	1.628	0	1	1	98%
39	TW	79	262	0	0	0	<23%
40	IS	72	NA	0	0	0	<23%

PCP: *Pneumocystis jirovecii* pneumonia; DIF: Direct Immunofluorescence; T-CD4+: T-CD4 count; VL: Viral load; IS: Induced sputum; BAL: Bronchoalveolar lavage; TW: Tracheal wash; SS: Spontaneous; 1 = positive; 0 = negative
